# The Agony of Choice—Where to Place the Wave of BCMA-Targeted Therapies in the Multiple Myeloma Treatment Puzzle in 2022 and Beyond

**DOI:** 10.3390/cancers13184701

**Published:** 2021-09-19

**Authors:** Irene Strassl, Martin Schreder, Normann Steiner, Jakob Rudzki, Hermine Agis, Tina Künz, Nino Müser, Wolfgang Willenbacher, Andreas Petzer, Peter Neumeister, Maria Theresa Krauth

**Affiliations:** 1 Division of Hematology with Stem Cell Transplantation, Hemostaseology and Medical Oncology, Department of Internal Medicine I, Ordensklinikum Linz, 4020 Linz, Austria; andreas.petzer@ordensklinikum.at; 2 First Department of Medicine, Klinik Ottakring, 1160 Vienna, Austria; martin.schreder@gesundheitsverbund.at (M.S.); nino.mueser@gesundheitsverbund.at (N.M.); 3 Division of Hematology and Medical Oncology, Department of Internal Medicine V, Medical University of Innsbruck, 6020 Innsbruck, Austria; normann.steiner@i-med.ac.at (N.S.); jakob.rudzki@tirol-kliniken.at (J.R.); tina.kuenz@student.i-med.ac.at (T.K.); wolfgang.willenbacher@tirol-kliniken.at (W.W.); 4 Division of Hematology and Hemostaseology, Department of Internal Medicine I, Medical University of Vienna, 1090 Vienna, Austria; hermine.agis@meduniwien.ac.at; 5 Oncotyrol, Center for Personalized Cancer Medicine, 6020 Innsbruck, Austria; 6 Division of Hematology, Medical University of Graz, 8036 Graz, Austria; peter.neumeister@medunigraz.at

**Keywords:** multiple myeloma, BCMA, CAR-T, bispecific antibodies, BiTEs^®^, antibody-drug conjugates (ADC), treatment selection

## Abstract

**Simple Summary:**

There is no doubt that immunotherapeutic approaches will change the current treatment landscape of multiple myeloma in the near future; in particular, a wave of BCMA-targeted therapies is currently entering clinical routine. Although the increasing availability of different therapeutic approaches is highly welcome, it also increases the daily challenges in clinical decision making if they all use the same target. Here, we provide a comprehensive summary of BCMA-targeted approaches in myeloma and aim to share some basic concepts in clinical decision making.

**Abstract:**

Since the introduction of first-generation proteasome inhibitors and immunomodulatory agents, the multiple myeloma (MM) treatment landscape has undergone a remarkable development. Most recently, immunotherapeutic strategies targeting the B cell maturation antigen (BCMA) entered the clinical stage providing access to highly anticipated novel treatment strategies. At present, numerous different approaches investigate BCMA as an effective multi-modal target. Currently, BCMA-directed antibody–drug conjugates, bispecific and trispecific antibodies, autologous and allogeneic CAR-T cell as well as CAR-NK cell constructs are either approved or in different stages of clinical and preclinical development for the treatment of MM. This armamentarium of treatment choices raises several challenges for clinical decision making, particularly in the absence of head-to-head comparisons. In this review, we provide a comprehensive overview of BCMA-targeting therapeutics, deliver latest updates on clinical trial data, and focus on potential patient selection criteria for different BCMA-targeting immunotherapeutic strategies.

## 1. Introduction

Multiple myeloma (MM) is a heterogenous disease, characterized by a malignant proliferation of clonal plasma cells. In 2020, the incidence of MM was 176,404 and the overall mortality was 117,077 worldwide (absolute numbers) [1]. Despite a markedly improved survival of patients throughout recent decades due to the development of new anti-myeloma agents, prognosis for patients with refractory disease is poor. Moreover, patients who are refractory to a proteasome inhibitor (PI), an immunomodulatory agent (IMiD), and a monoclonal antibody have a median overall survival (OS) of less than a year [2,3]. Therefore, new treatment strategies using alternative targets are urgently needed.

**Figure 1:**
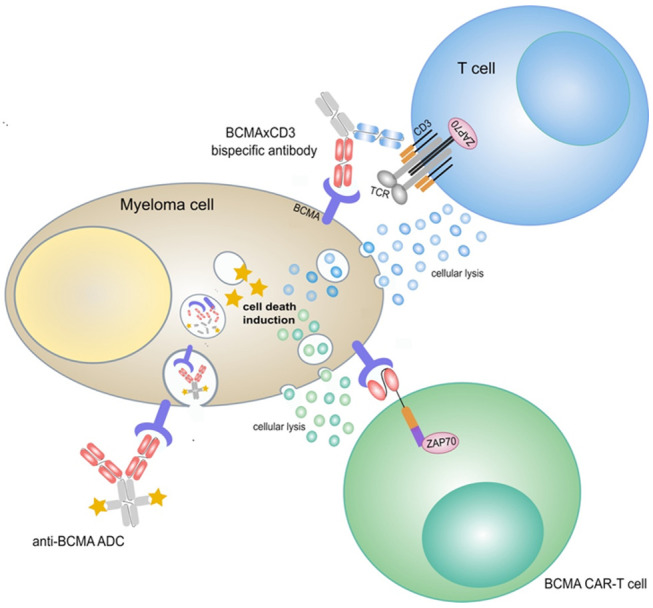
Schematic illustration of BCMA-targeted immunotherapeutic constructs. ADC, antibody drug conjugate; BCMA, B cell maturation antigen; CAR, chimeric antigen receptor; TCR, T cell receptor.

B cell maturation antigen (BCMA), a transmembrane glycoprotein member of the tumor necrosis factor receptor superfamily, is expressed on the surface of normal and malignant plasma cells and mature B cells, with minimal expression on other tissues. BCMA has a crucial role in the survival of malignant plasma cells through the regulation of maturation, differentiation and activation of survival and proliferation pathways [4,5,6]. Thus, BCMA seems to be an ideal target in the treatment of multiple myeloma and is therefore extensively studied. The most clinically advanced BCMA-targeting treatment modalities are bispecific antibody constructs, antibody–drug conjugates (ADCs) and chimeric antigen receptor (CAR) T cells. Figure 1 shows a schematic illustration of BCMA-targeted immunotherapeutic constructs.

In this review, we give an overview of novel immunotherapeutic approaches in MM, with particular focus on BCMA-targeted therapies. Moreover, we aim to share our thoughts on key selection criteria for the future stratification of MM patients to different BCMA-targeting treatment options.

## 2. CAR-T Cells

Chimeric antigen-receptor T cells are immune cells genetically modified to target antigen-expressing tumor cells. Chimeric antigen receptors are fusion proteins containing an antigen-recognition moiety, a T cell activation domain, and a costimulatory domain. The costimulatory domain in addition to the CD3ζ intracellular signaling domain leads to better clinical activity through an enhanced likelihood of T cell proliferation [7,8].

CAR-T cells targeting CD19 showed impressive responses in different hematologic malignancies and are approved for the treatment of relapsed and refractory (r/r) acute lymphoblastic leukemia, r/r diffuse large B cell lymphoma, and r/r mantle cell lymphoma [9,10,11,12]. Through the promising results of targeting CD19, other targets are also intensively investigated.

The first anti-BCMA CAR-T cells were synthesized in 2013 and showed activity in multiple myeloma cell lines [6]. Since then, several BCMA-targeting CAR-T cell constructs have been developed and are currently explored in clinical trials. In 2016, the first clinical results of anti-BCMA CAR-T cells were published with promising responses in individual patients with refractory disease and high tumor burden [13]. To date, idecabtagene vicleucel (ide-cel, bb2121) is the only approved CAR-T cell product for the treatment of relapsed and refractory multiple myeloma (RRMM), but other products will presumably follow within the next year. Furthermore, trials with anti-BCMA CAR-T cells in earlier stages of the disease are ongoing and CAR-T cells targeting other multiple myeloma antigens such as CD38, CD138, and SLAMF7 are also being explored.

In general, CAR-T cells are typically generated from T cells collected from the patient via leukapheresis and then modified and expanded ex vivo. As the manufacturing process usually lasts several weeks, patients may receive bridging therapy to maintain disease control before CAR-T cell infusion. Most patients also undergo a conditioning lymphodepletion chemotherapy to reduce endogenous levels of lymphocytes before reinfusion of the CAR-T cell product. The most common side effects across all CAR-T cell therapies are cytokine release syndrome (CRS), neurotoxicity, cytopenia, and infections. CRS is initiated by T-cell activation and results from a massive release of inflammatory cytokines, particularly interleukin 6 (IL-6) and interferon gamma (IFN-γ) [14,15,16]. CRS can cause a wide variety of clinical symptoms, usually beginning with fever, followed by hypotension, tachycardia, or respiratory insufficiency [14]. Neurotoxicity can also manifest itself in several ways, up to seizures and brain edema in rare cases. A typical early sign of neurotoxicity is altered handwriting, which is why a handwriting sample is included in daily cognitive testing [17]. BCMA-targeting CAR-T cells showed no surprising off-target toxicity in clinical studies so far [7].

### 2.1. Idecabtagene Vicleucel

Ide-cel or bb2121 is the first FDA- and EMA-approved BCMA-targeting CAR-T cell construct for the treatment of relapsed and refractory multiple myeloma. The first in-human phase I/II CRB-401 study demonstrated good tolerability and promising efficacy in patients with RRMM [18]. The updated results showed deep and durable responses with ide-cel in triple-class exposed patients with a median PFS of 8.8 months and a median OS of 34.2 months across all treated patients (*n* = 62). Half of the ongoing responders achieved a duration of response (DOR) of more than two years. All patients with at least a CR who had a qualified assessment achieved MRD negativity by NGS (sensitivity ≤10^−4^ nucleated cells). Efficacy and safety are consistent with prior reports and highlight a favorable clinical benefit–risk profile for ide-cel at target dose levels ≥150 × 10^6^. The CRS rate was 75.8% with 6.5% grade 3 CRS, and all others were grade 1 or 2. Time to first onset was 2 days and the median duration was 5 days. Neurotoxicity was less common, with 35.5% overall and mostly grade 1 [19]. The pivotal phase 2 KarMMa study demonstrated similar results. A total of 128 patients were treated with ide-cel and included a high proportion of high-risk patients, namely 35% with high-risk cytogenetics and 39% with extramedullary disease. The longest responses were achieved at the highest dose level of 450 × 10^6^ with a PFS of 12.2 months [20]. At a median follow-up time of 13.3 months, 33% of the patients had a complete response or better. In this case, of heavily pretreated patients, the most exciting fact was that an MRD negativity of <10^−5^ nucleated cells were confirmed in 26% of all patients treated and 79% of 42 patients with a CR or better [21]. In subgroup analyses of the KarMMa, study responses of high-risk patients were promising, the median DOR was 9.2 months, and the median PFS 7.5 months in this difficult-to-treat population. Additionally, older patients ≥70 years (16% of all treated patients) responded well, and no new safety signals were observed [22,23].

To obtain longer persistence and function of CAR-T cells, bb21217 was developed. bb21217 uses the same CAR molecule as bb2121 but is cultured with a PI3K inhibitor to enrich for T cells displaying a memory-like phenotype. The last data update of the phase 1 CRB-402 study showed long-term CAR-T cell persistence in 6 of 11 evaluable patients. ORR at the recommended phase 2 dose (RP2D) was 84% [24].

There are several ongoing trials addressing different types of patients and different lines of therapy, such as KarMMa-2, a phase 2 trial studying high-risk MM with progression within 18 months after first line therapy or inadequate response to ASCT (NCT03601078). Another study, KarMMa-3, is comparing standard of care (SOC) in triple class refractory patients with ide-cel (NCT03651128). The KarMMa-4 trial is attempting to demonstrate efficacy in newly diagnosed multiple myeloma (NDMM) with R-ISS stage III disease per IMWG criteria (NCT04196491). Finally, KarMMa-7 is a phase 1/2 study to determine the safety, tolerability, and efficacy of bb2121 in combination with other therapies in patients with RRMM (NCT04855136).

### 2.2. Ciltacabtagene Autoleucel

The next CAR-T cell product on the horizon is Ciltacabtagene autoleucel (cilta-cel, also known as JNJ-4528 or LCAR-B38M in China). Cilta-cel contains two BCMA-targeting single heavy chain domains and a 4-1BB costimulatory domain besides the typical T cell activating domain [25].

The first clinical results of this CAR-T cell construct were presented from the LEGEND-2 study from China. The ORR was 88% and responses were deep and durable with low CAR-T cell doses. Overall, a median PFS of 20 months was reached (*n* = 57); OS at 18 months was 68% [26]. The CARTITUDE-1 trial also investigated the efficacy and safety of cilta-cel in RRMM. Patients were eligible after at least three prior regimens including a PI, an IMiD, and an anti-CD38 antibody. In this heavily pretreated patient cohort, 87.6% were triple-class refractory and 42.3% penta-class refractory. Patients received a median of six prior lines. At the latest update at EHA 2021 for 97 treated patients, ORR was 97.9% with an impressive sCR rate of 80.4%. Remarkably, the sCR rate increased from 67% to 80.4% in the six months since the last data update at ASH 2020. Almost all evaluable patients were MRD negative (91.8%; MRD at 10^−5^). The 18-month PFS was 66% and 18-month OS resulted in 80.9%. The most common AEs were cytopenia and CRS in 95% of patients each. CRS was mostly grade 1 and 2; 5% of patients developed higher-grade CRS and one patient died. The median time to CRS onset was 7 days, which is longer than in other products and is attributed to the lower administered cell dose with later peak expansion of CAR-T cells. Tocilizumab was used in 69.1% and corticosteroids in 21.6% of patients. CRS resolved in 98.9% of patients within 14 days of onset. Higher-grade neurotoxicity (grade ≥3) occurred in 10.3%. Unlike in other CAR-T cell trials, neurotoxic events were divided in ICANS (2.1% grade ≥3) and other neurotoxicities (9.3% grade ≥3), which were defined as events with later onset after a period of recovery from ICANS. These quite atypical neurological symptoms could be prevented by a potent prior salvage therapy regimen and extended monitoring with early therapy of CRS and ICANS [27,28,29].

Recently, the first results from cohort A of the CARTITUDE-2 study were presented (NCT04133636). In this phase 2 study, cilta-cel is investigated in patients in different MM settings. Cohort A is eligible for patients with progressive disease after 1–3 lines of therapy and who are lenalidomide refractory. ORR and CR rates were similar to CARTITUDE-1. No progression of disease was observed at a median follow up of 5.8 months [30]. Furthermore, cilta-cel is being investigated in the phase 3 CARTITUDE-4 study comparing CAR-T cell therapy versus Pomalidomide, Bortezomib and Dexamethasone (PVd) or Daratumumab, Pomalidomide and Dexamethasone (DPd) in patients with relapsed and Lenalidomide-refractory MM (NCT04181827). The CARTITUDE-5 study will be the first study exploring cilta-cel in newly diagnosed elderly multiple myeloma patients (NCT04923893).

### 2.3. ALLO-715

Another interesting but preliminary approach in adoptive T cell transfer are allogeneic CAR-T cells: ALLO-715 is a genetically modified allogeneic anti-BCMA CAR-T cell product in which the TCR alpha constant gene is disrupted to reduce the risk of graft versus host disease (GvHD). This CAR-T cell construct will be concomitantly administered with an anti-CD52 monoclonal antibody (ALLO-647) for selective and prolonged host lymphodepletion. The greatest advantage in this technique is the rapid availability of the transgenic CAR-T cells. The first presented result of a phase 1 dose escalation trial showed dose-dependent activity in heavily pretreated patients. Altogether, about 60% (six patients) achieved ORR and 40% a VGPR+ (sCR, CR or VGPR). ALLO-715 was well-tolerated at all dose levels. A very remarkable aspect was that no GvHD and no neurotoxicity were described. The safety data refer to CRS up to grade 2 as a maximum grading. All other side effects were comparable to other CAR-T cell constructs. A total of 90% of study patients were treated within 5 days of study enrollment, which underscores the fast availability of an allogeneic construct compared to autologous CAR-T cells [31].

### 2.4. Other BCMA-Targeting CAR-T Cell Constructs

Of the several other anti-BCMA CAR-T cell constructs, P-BCMA-101 is of special interest as it is the first BCMA-targeting construct that uses a gene transfer system without a viral vector (piggyBac^®^ DNA Modification System). This CAR-T cell construct comprises a safety switch for rapid CAR-T cell elimination in case of severe CRS. The ongoing phase 1/2 PRIME study includes multiple exploratory cohorts with different drug combinations. During phase 1, the manufacturing process was modified with Nanoplasmid to improve transposition. P-BCMA-1010 with Nanoplasmid demonstrated an ORR of 66.7% (*n* = 6). CRS occurred in 17% (*n* = 53), all grade 1 and 2. The safety switch was not needed so far [32].

Table 1 lists clinically investigated CAR-T cell constructs for the treatment of multiple myeloma.

## 3. Bispecific Antibodies

Redirecting T cells to tumor cells by means of bispecific antibodies (BsAbs) is an attractive strategy because these antibody constructs are available off-the-shelf and are relatively easy to administer. The T cell-recruiting BsAbs activate T cells by binding CD3ε in the T-cell receptor complex, which leads to T cell activation independent of major histocompatibility complex (MHC) restriction. Furthermore, BsAbs have the ability of T cell activation in the absence of co-stimulation [38,39]. Because formation of the cytolytic synapse is independent of standard antigen recognition and co-stimulation, immune escape mechanisms of tumor cells are evaded [8]. T cell activation finally leads to tumor cell lysis through the release of perforins and granzymes which induce apoptosis [40,41].

**Figure 2:**
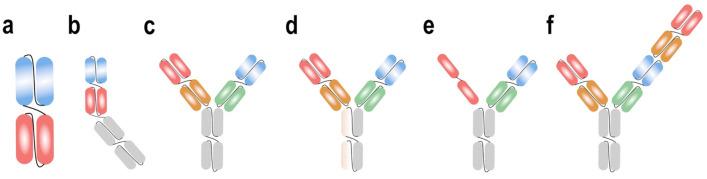
Selected bispecific T cell engaging constructs targeting BCMA and CD3. BCMA-targeting regions are colored red, CD3-targeting regions are blue. Fc regions are colored grey. (**a**) Bispecific T cell engager (BiTE^®^, AMG420). (**b**) Half-life extended bispecific T cell engager (HLE BiTE^®^, AMG701). (**c**) Bispecific antibody, IgG4 Fc region (REGN5458, Elranatamab). (**d**) Bispecific antibody, IgG4 Fc region (DuoBody^®^, Teclistamab). (**e**) Bispecific antibody, IgG4 Fc region, dual BCMA binding domains (TNB-383B). (**f**) Bispecific antibody, IgG1 Fc region, bivalent anti-BCMA arm (CC-93269).

In general, two distinct groups of BsAbs can be distinguished based on the presence or absence of an Fc domain. Apart from the longer half-life, the Fc region induces antibody-dependent cell-mediated cytotoxicity (ADCC) and mediates complement-dependent cytotoxicity, if not silenced. Constructs without an Fc domain are known as bispecific T cell engagers (BiTEs^®^) and consist of two different single-chain variable fragments [42,43]. The final antibody can be made of various known fragments and leads to a great diversity of antibody constructs (Figure 2).

The main toxicities of BsAbs are CRS, neurotoxicity, cytopenia and infections. Usually, CRS occurs only during step up dosing or after the first administration of a BsAb [44]. Another challenging fact is tumor cell resistance caused by T cell exhaustion, antigen escape, or an immunosuppressive microenvironment. Different strategies to overcome these problems have been developed and are currently being investigated, for example combined targeting of several antigens or combination of BsAbs with other immunomodulatory therapeutics [38,45,46].

BsAbs-targeting multiple myeloma cells have demonstrated encouraging results in preclinical studies in vitro, in mouse xenograft models, and in cynomolgus monkeys [47,48,49,50,51,52]. To date, more than 10 different BsAbs-targeting MM antigens have been clinically investigated, most of them directed to BCMA and CD3 with promising clinical data from phase 1/2 studies (Table 2) [45].

### 3.1. AMG420 and AMG701

The BCMA/CD3 BiTE^®^ AMG420 is comprised of two single-chain variable fragments (scFvs) and was the first-in-class bispecific construct in MM [47]. The first-in-human study showed an overall response rate (ORR) of 70% (*n* = 7 of 10) at the maximum tolerated dose (MTD) of 400 μg/d. Five patients achieved an MRD-negative complete remission (CR) (MRD at 10^−4^). Serious adverse events (SAE) included infections, polyneuropathy and edema. CRS rate was 38% and mostly grade 1 or 2; only one patient experienced a CRS grade 3. There were no higher-grade central nervous system (CNS) toxicities. Long-term follow-up data have been presented for 23 of the 42 enrolled patients. Ten of the 23 patients responded. The PFS of the responders was 23.5 months. Because of logistical challenges due to continuous intravenous infusion, further development has been halted [53,63].

AMG701 is a half-life extended BiTE^®^ comprised of the same two scFv regions as AMG420 and an additional Fc region which enables once-weekly dosing [48]. Initial results of an ongoing phase 1/2 study showed promising response rates at higher doses of AMG701. At data cutoff, 75 patients received AMG701 as weekly intravenous infusion in 4-week cycles. Patients were heavily pretreated with a median of six prior lines of therapy; 68% were triple refractory to a PI, an IMiD, and an anti-CD38 antibody, and 27% of patients had extramedullary disease. As expected, cytopenia was common; 43% of patients had anemia, 23% neutropenia and 20% thrombocytopenia. The most common non-hematological AE was CRS (61%); 7% of patients experienced grade 3 CRS, and all others were grade 1 or 2. All CRS were reversible with corticosteroids and tocilizumab, with a median duration of two days. Other AEs included diarrhea (31%), fatigue (25%), and fever (25%). The response rate was 36% (16/45) at doses of 3–12 mg. With further dose escalation, the response rate in the last evaluated cohort (*n* = 6) was 83%. Across all patients, responses included four stringent CRs (3/3 tested patients MRD-negative). Responses occurred fast, the data for the duration of response were not mature, and maximum duration of response was 23 months [54]. In addition, the ongoing study will include a sequential dose escalation part to identify the recommended phase 2 dose (RP2D) of AMG701 in combination with pomalidomide, with and without dexamethasone (NCT03287908). The rationale for combination therapy with an IMiD is based on preclinical studies, which showed enhanced anti-tumor activity with combination therapy in vitro and in xenograft mouse models [64].

### 3.2. Teclistamab

Teclistamab (formerly known as JNJ-64007957 or JNJ-7957) is a humanized IgG-4-bispecific DuoBody^®^ antibody targeting BCMA and CD3 [51]. The phase 1 first-in-human study consisted of two parts with weekly intravenous or subcutaneous administration of Teclistamab to assess RP2D. The first presented results for intravenous dosing showed a 67% ORR at the highest tested dose of 270 μg/kg (*n* = 12) [55]. Overall, 157 patients were enrolled; 84 of them received intravenous and 73 subcutaneous Teclistamab in different dosing groups. Eligible patients had to be relapsed/refractory (RR) or intolerant to established MM therapies. The median number of prior lines was six, 33% of patients had high-risk cytogenetics, 82% were triple-class refractory, 39% were penta-drug refractory, and 90% were refractory to the last line of therapy. In addition to hematological AEs, the most common treatment-related AE was CRS in 57% of patients with a maximum grade 2. Tocilizumab was administered in 24%, steroids in 15%. Of note, CRS occurred later and lasted longer with subcutaneous administration with a median time to onset of two days and a duration of two days versus one day each with intravenous dosing. Neurotoxicity was observed in 4% of patients, including two higher-grade events with intravenous dosing. Other common AEs included infections, pyrexia, diarrhea, cough, fatigue, back pain, and headache [56]. Most active doses were 270–720 μg/kg IV and 720–3000 μg/kg SC. ORR in this dosing groups was 69% (47/68) with at least VGPR in 59% and at least CR in 26% [57]. Forty patients received the RP2D of 1500 μg/kg SC. The ORR in this group was 65%, with 40% achieving at least a CR. The median duration of follow-up for all 40 patients treated at the RP2D was 6.1 months. Of the 26 evaluable patients across all doses and cohorts, 18 had MRD-negative CR/sCR (69%). All evaluable patients in the RP2D cohort (*n* = 6) achieved MRD negativity (<10^−5^) [56]. Responses were durable and deepened over time, and 85% (22/26) of responders at the RP2D of 1500 μg/kg SC remained on therapy after a median follow up of 7.1 months [58]. The efficacy of Teclistamab in the RP2D of 270 μg/kg IV and 1500 μg/kg SC is currently evaluated in a phase 2 study (NCT04557098). Furthermore, another study investigates subcutaneous Daratumumab in combination with either Teclistamab or Talquetamab with or without pomalidomide (NCT04108195). The rationale of a combination of Daratumumab with Teclistamab is based on preclinical data, which showed enhanced MM cell lysis with the direct combination. Furthermore, the activity of Teclistamab was significantly enhanced in bone marrow samples of Daratumumab-pretreated patients and tumor cell lysis was superior when T cells obtained from patients treated with Daratumumab were used [65].

### 3.3. REGN5458

REGN5458 is a fully human BsAb that binds to BCMA and CD3 [50]. The safety and efficacy data of 49 patients from an ongoing phase 1 study were recently presented. Patients were eligible after at least three prior lines of therapy, including a PI, an IMiD, and an anti-CD38 antibody. The BsAb was administered weekly, followed by a maintenance phase with biweekly doses. All included patients were triple-refractory, 57% were penta-refractory. CRS occurred in 39%, and no grade 3 or higher events were observed. As in other studies with BsAbs, hematologic AEs, infections, fatigue, and myalgias were common. Mild neurotoxicity appeared in 12% of patients. No new safety signals were seen. Responses were dose-dependent and occurred early. In the highest tested dose level of 96 mg, ORR was 62.5% (*n* = 8), all VGPR. Overall, 95% (18/19) of responders achieved VGPR or better, and 42% (8/19) had CR or sCR. MRD status was evaluated in seven patients; 57% were MRD-negative (<10^−5^) [59]. Another phase 1 trial was initiated for REGN5459, a similar BCMA/CD3 BsAb, which has different binding characteristics (NCT04083534).

### 3.4. TNB-383B

TNB-383B is a fully human triple-chain BCMA × CD3 BsAb with a unique anti-CD3 moiety for target lysis with minimal cytokine release and two anti-BCMA moieties [52]. Data of 58 patients from the ongoing first-in-human study are available. TNB-383B was administered intravenously every 3 weeks without step-up dosing. Patients enrolled had received a median of six prior lines of therapy, 64% were triple-class refractory and 34% penta-drug refractory. Safety data were comparable to the other described studies. No higher-grade CRS was observed. One patient developed grade 3 neurotoxicity (confusion). At the highest evaluated doses of 40–60 mg, ORR was 80% (*n* = 15), 73% achieved VGPR or better. A total of 81% (22/27) of responding subjects have ongoing responses [60].

### 3.5. Elranatamab

Elranatamab (formerly known as PF-06863135) is a humanized anti-BCMA/CD3 BsAb with an IgG2a backbone [49]. Early clinical data for intravenous and subcutaneous administration showed activity in patients with RRMM. With weekly intravenous dosing, the best response in the early-dose escalation phase (*n* = 17) was minimal response in 6% and stable disease in 35%. A total of 29% of patients had received another BCMA-targeted therapy prior to study enrollment [66]. Subcutaneous dose escalation was subsequently initiated to achieve a more favorable therapeutic window. Among 30 patients in the SC dose escalation cohort, responses were seen at doses ≥215 μg/kg in 20 patients. ORR in these heavily pretreated patients was 70% with a CR/sCR rate of 30% (*n* = 20). Six patients received the RP2D of 1000 μg/kg SC; the confirmed ORR was 83.3%. Three out of four patients with prior BCMA-directed therapy responded, two of them achieved VGPR and one CR. The probability of responders being event-free at 6 months was 92.3%. CRS occurred in 73.3% of patients, with a maximum grade of 2. CRS onset was observed early, after a median of 1 day, and lasted for a median of 2 days. Tocilizumab was administered in 30% and steroids in 10% of patients. No higher-grade neurotoxicity was observed in this cohort [61].

### 3.6. CC-93269

CC-93269 is an asymmetric dual-arm, human IgG1-based BsAb with one CD3 and two BCMA binding sites [67]. Patients had received at least three prior regimens. CC-93269 was administered by IV weekly until cycle 3, biweekly from cycle 4 to 6, and every 4 weeks thereafter. In the most recent study update, data from 30 patients were presented. Overall, the safety profile was comparable with other published data for bispecifics. CRS was reported in 77% of patients, in 27% at least grade 3. One patient died during the study in the setting of CRS. At the highest dose of 10 mg (*n* = 9), ORR was 89%, including 44% CR/sCR. 92% of responding patients (*n* = 13) achieved MRD negativity (<10^−5^) [62].

### 3.7. Other Constructs

HPN-217 is a BCMA-targeting trispecific T cell activating construct (TriTAC) containing three humanized antibody-derived binding domains, targeting BCMA, CD3, and albumin (for half-life extension). The phase 1 study is recruiting, and the first clinical data are awaited [68].

Other BCMA-targeting constructs in preclinical development are bispecific or trispecific NK cell-based antibodies. In comparison to T cell-based antibodies, NK cell-based constructs show less inflammatory cytokine secretion in preclinical models. Examples for NK-cell BsAbs are AFM26 and CTX-8573, which showed preclinical activity in vitro and in vivo [38,39].

## 4. Antibody Drug Conjugates

Antibody-drug conjugates (ADCs) represent a novel class of immunotherapeutics which are increasingly used in the treatment of hematologic malignancies and solid cancers [69,70,71,72]. They consist of a monoclonal antibody that is linked to a cytotoxic drug and is internalized upon binding to its target antigen on the cell surface, thus delivering the toxic payload directly to the tumor cell (Table 3).

### 4.1. Belantamab Mafodotin

Belantamab mafodotin (GSK2857916, belamaf) is a first-in-class ADC for the treatment of multiple myeloma featuring the humanized anti-BCMA antibody J6M0 with a defucosylated Fc portion and monomethyl auristatin F (MMAF, mafodotin) as its effector molecule [73]. In preclinical models, the compound competes for binding to BCMA with BAFF and APRIL and inhibits ligand induced NFkB signaling. Once internalized, MMAF is cleaved and retained inside target cells where it causes dose- and time-dependent G2/M cell cycle arrest followed by apoptosis [73,77]. In addition, the compound induces enhanced antibody-dependent cell lysis due to its modified Fc moiety as well as macrophage-mediated phagocytosis of MM cells [73].

The first-in-human, open-label phase 1 study DREAMM-1 enrolled 73 patients with refractory MM and previous exposure to alkylators (including an autologous stem cell transplant, if eligible), IMiDs, and PIs. The dose-escalation phase (part 1, *n* = 38) reported corneal events (53%), nausea (49%), fatigue (47%), and thrombocytopenia (42%) as the most common side effects [78]. No dose-limiting AEs were observed and a formal MTD was not reached, but ocular toxicities, which are frequently observed in association with the administration of ADCs [79], tended to be more severe at higher dose levels. Based on these observations and a lack of clinical activity in patients treated at 2.5 mg/kg, a recommended phase 2 dose of 3.4 mg/kg was determined. In the dose expansion phase (part 2), 35 patients with heavily pretreated MM (40% with >5 prior therapies) received belamaf at 3.4 mg/kg as intravenous infusion every three weeks for a maximum of 16 cycles [78]. Totals of 94% and 97% of patients were refractory to PIs and IMiDs, respectively, and 40% had daratumumab refractory disease. Again, keratopathy resulting in blurred vision, dry eyes, and photophobia was the single most common adverse event, affecting 69% of patients. Infusion-related reactions occurred with the first dose in 29% of patients and were mainly grade 1 and 2, and no new toxicities emerged. Dose reductions and dose delays or interruptions were required in 66% and 71% of patients, respectively. AEs leading to permanent discontinuation of the drug occurred in only four cases (11%). The overall response rate in this cohort was 60% and five patients achieved a complete remission. Responses were rapid with a median time to first response of 1.2 months. Progression-free survival was 12.0 months overall, 7.9 months in double refractory patients, and 6.8 months in those with prior daratumumab. 

DREAMM-2 was designed as a two-arm, randomized, open-label phase 2 study to evaluate the efficacy and safety of belamaf administered every 3 weeks both at the recommended phase 2 dose of 3.4 mg/kg and a lower dose of 2.5 mg/kg due to the frequent dose modifications in the phase 1 trial [80]. The study enrolled 196 patients with relapsed or refractory MM after three or more lines of therapy who were refractory to PIs and IMiDs and refractory or intolerant to an anti-CD38 monoclonal antibody. A total of 83% of patients had received >four prior therapies; high-risk cytogenetics and extramedullary lesions were present in 45% and 20%, respectively. The median number of treatment cycles was three in both treatment arms (*n* = 97 in the 2.5 mg/kg group, *n* = 99 in the 3.4 mg/kg group). An overall response was achieved in 30/97 patients (31%) in the 2.5 mg/kg cohort and 34/99 patients (34%) in the 3.4 mg/kg cohort. The proportion of patients reaching a very good partial response or better was identical at both dose levels (19% vs. 20%), as was the complete response rate (three patients in each cohort). The median duration of response (DoR) and overall survival were not reached at 6 months of follow-up in the primary analysis. With an extended follow-up of 13 months, the median DoR and OS estimate was 11 months and 13.7 months, respectively, in the 2.5 mg/kg cohort compared to 6.2 months and 13.8 months, respectively, in the 3.4 mg/kg cohort [81]. Among patients treated at a dose of 2.5 mg/kg, there was no apparent difference in efficacy and occurrence of adverse events between subgroups with 3–6 versus ≥ 7 prior lines of therapy [82]. In prespecified subgroups of patients with moderate renal insufficiency or high-risk cytogenetics, depth and durability of responses were comparable to those seen in the overall population [83]. However, belamaf seemed to confer less benefits in patients with extramedullary disease. In terms of safety, the most frequent AEs were corneal events seen in 71% and 77%, respectively, in both treatment arms. The internalization of belamaf by corneal epithelial cells through macropinocytosis and subsequent apoptosis is thought to induce the typical microcyst-like epithelial changes (MECs) that appear as bilateral, diffuse, microcyst-like lesions on slit-lamp photography [84]. Keratopathy was the most frequent cause for dose reductions (23% and 27%) and delays (47% and 48%, starting at week 4), but was a rare cause for permanent discontinuation (one and three patients). Twenty-two patients in each treatment arm had definite worsening of best-corrected visual acuity (BCVA) at the end of treatment, with no reports of complete permanent vision loss. Most patients recovered from their first keratopathy event (77%) or from clinically meaningful BCVA deterioration (82%) [85]. In an ocular substudy of DREAMM-2 (*n* = 30), corticosteroid eyedrops failed to mitigate eye toxicity [80]. Preservative-free lubricating eyedrops administered at least four times daily prior to and throughout the treatment period remain the only recommended pharmacotherapy along with frequent eye examinations and dose modification in the event of worsening symptoms. Other adverse events in DREAMM-2 were more common in the 3.4 mg/kg group, such as thrombocytopenia (grade 3/4 in 33% vs. 20%) and pneumonia grade 3 or worse (11% vs. 4%). Dose modifications were more frequent and median dose intensity was significantly lower in than the intended dose for the 3.4 mg/kg group. Based on these data, belamaf at a dose of 2.5 mg/kg administered every three weeks was approved by both the FDA and EMA in 2020. A lyophilized formulation of the drug is intended for future clinical use instead of the liquid–frozen preparation, both of which showed comparable efficacy and safety data [86].

Combinations with both standard and novel treatments are currently being explored. A phase 1 trial reports a high efficacy of belamaf in combination with pomalidomide/dexamethasone in 35 patients, but frequent dose holds due to keratopathy at a dose of 2.5 mg/kg [87]. Alternative dosing schedules are under investigation and DREAMM-8 will compare the above-mentioned combination with bortezomib/pomalidomide/dexamethasone in patients with ≥1 prior therapies in a randomized phase 3 design [88]. Belamaf plus bortezomib/dexamethasone has a high response rate of 78% in heavily pretreated patients [89]. DREAMM-7 is currently enrolling patients with relapsed/refractory MM after ≥ 1 prior line of treatment to receive bortezomib/dexamethasone either with belamaf or daratumumab in a randomized phase 3 study [90]. Finally, DREAMM-5 is a phase 1/2 platform to explore the efficacy and safety of belamaf combined with an OX40 agonist, a gamma-secretase inhibitor, and a PD-1 blocker, among others [91].

### 4.2. Other BCMA-Targeting ADCs

Recently, novel ADCs targeting BCMA have entered clinical development. MEDI2228 uses an antibody domain that preferentially targets membrane-bound versus soluble BCMA, a cleavable linker and a DNA cross-linking pyrrolobenzodizepine (PBD) dimer as its payload [74]. As of 16 October 2020, 82 patients with refractory MM after treatment with PIs, IMiDs, and moAbs have been enrolled in the first-in-human, open-label phase 1 trial (NCT03489525) [92]. In the dose-escalation phase (*n* = 41), dose-limiting thrombocytopenia occurred in two patients at 0.20 mg/kg. An additional 41 patients, 56% of whom had triple-class refractory disease, were included in the MTD expansion cohort, and treated with MEDI2228 at 0.14 mg/kg every 3 weeks. Early onset photophobia was seen in 58.5% of patients (17.1% grade 3) after approximately 2–3 cycles which was not associated with keratopathy and was a frequent cause for drug discontinuation. Symptoms improved in 9/24 patients (37%) and completely resolved in four patients, but follow-up was limited. Further PBD class toxicities included a grade 1/2 rash (31.7%), thrombocytopenia (31.7%, grade 3/4 24.4%), pleural effusions (24.4%, grade 1/2 in all but one patient) and increased GGT (24.4%, grade 3/4 19.5%). MEDI2228 demonstrated clinical efficacy across all dose levels with an overall response rate of 66% at 0.14 mg/kg and a median time to response of 2.1 months. The median number of treatment cycles was three and the duration of response was 5.9 months, but this analysis was impacted by loss of patients to follow-up. Further expansions cohorts at 0.14 mg/kg with alternative dose and schedule to mitigate eye toxicities are currently being explored.

The RNA-polymerase inhibitor amanitin serves as the cytotoxic moiety in HDP-101, another BCMA-targeting ADC with potent activity in preclinical models [75]. A first-in-human phase 1/2a trial is expected to open recruitment in 2021 (NCT04879043) [93].

## 5. Drug Targets beyond BCMA in Clinical Development

### 5.1. FcRH5

The FcRH gene family members are Ig superfamily type I membrane proteins and are expressed only in the B-cell compartment. Fc receptor-homolog 5 (FcRH5) is found on naïve and memory B cells and on plasma cells [94]. As compared to normal plasma cells, FcRH5 expression is higher in multiple myeloma cells, and therefore FcRH5 seems to be a reliable target for the treatment of multiple myeloma [95,96]. The first drug in clinical development targeting FcRH5 was DFRF4539A, an antibody–drug conjugate linking a humanized IgG1 monoclonal antibody against FcRH5 with monomethyl auristatin E. As the phase I study was unsuccessful due to limited activity and adverse events, further development was stopped [97]. A more promising research approach is bispecific antibodies. An anti-FcRH5/CD3 bispecific antibody demonstrated anti-myeloma activity in vitro and in a model with cynomolgus monkeys [96]. The first results of a phase I trial of the humanized IgG-based T-cell engaging bispecific antibody Cevostamab (formerly known as BFCR4350A) were presented by the end of 2020. Cevostamab was administered by IV infusion in 21-day cycles. At data cut-off, 53 patients had been enrolled with a median number of six prior treatment lines, including prior anti-BCMA therapy in 21%. Responses were observed at doses ≥3.6/20 mg and ORR was 53% (18/34) in this patient cohort, with 18% of patients achieving CR or better. So far, eight patients had a duration of response longer than 6 months. CRS was the most common adverse event in 76% of patients; one patient had grade 3 CRS, and all other cases were grade 1 or 2 [98].

### 5.2. GPRC5D

Another potential target to circumvent antigen escape through reduced surface expression of BCMA is G-protein-coupled receptor family C group 5 member D (GPRC5D). GPRC5D is overexpressed in poor-risk myeloma, whereas only low expression is detected in normal tissues, except in hair follicles [99,100].

A model that investigated CAR-T cells targeting BCMA and GPRC5D simultaneously showed that targeting GPRC5D can prevent a BCMA escape-mediated relapse [101]. A phase I clinical trial currently investigates GPRC5D-directed CAR-T cell therapy (MCARH109) in relapsed/refractory multiple myeloma, including patients who have received prior BCMA-targeted therapies (NCT04555551). Dual-targeted CAR-T cell constructs are also in development, and a combination of BCMA and GPRC5D showed promising activity in preclinical models [102].

Talquetamab is a first-in-class humanized DuoBody^®^ with an IgG4 backbone that binds to GPRC5D and CD3 to redirect T-cells to myeloma cells and induces killing of GPRC5D positive cells [103]. The first-in-human MonumenTAL-1 study evaluates the efficacy and safety of Talquetamab in patients with multiple myeloma, relapsed/refractory, or intolerance to established therapies. So far, data from 184 patients were presented; 102 in the IV cohorts, and 82 in the SC cohorts. The median number of prior therapies was six, most patients were triple-class refractory and refractory to the last line of therapy. In addition to hematologic AEs, the most common non-hematologic AE was CRS with 73% at the RP2D in the SC cohort (*n* = 30), including one grade 3 CRS, two grade 2 and all others grade 1. Tocilizumab was used in 60% of these patients. Neurotoxicity was observed in four patients with SC dosing (all grade 1/2). Skin-related AEs were seen in 67% of SC treated patients, nail disorders in 21%, and infections in 37%. ORR at most active IV doses was 67% (12/18), and ORR at the RP2D of 405 μg/kg SC once weekly was 70.0% (21/30). Responses were durable and deepened over time, and 81% of responders were continuing on treatment after a median follow up of 6.3 months [104,105]. A phase 2 expansion study of Talquetamab at the RP2D is recruiting (NCT04634552).

### 5.3. SLAMF7

Signaling lymphocytic activation molecule F7 (SLAMF7, also known as CS-1 or CD319) is a well-known target in multiple myeloma. SLAMF7 is highly expressed on myeloma cells with minimal expression on healthy tissue [106]. The phase III ELOQUENT-2 and ELOQUENT-3 trials resulted in the approval of the fully humanized monoclonal antibody Elotuzumab in combination with lenalidomide or pomalidomide and dexamethasone [107,108].

Several new constructs targeting SLAMF7 have been investigated, most of them in preclinical or in early clinical development. While an ADC failed to show significant efficacy [109] and redirecting T-cells against a self-antigen may appear difficult, preclinical models demonstrated promising efficacy of SLAMF7-directed CAR-T cells [110] and combinatorial targeting with hemibodies addressing CD38 and SLAMF7 [46]. SLAMF7 or CS-1 CAR-T-cells are currently being tested in several phase I trials (NCT03710421, NCT04541368, NCTO04499339, NCT04142619).

### 5.4. CD38

CD38 is a transmembrane glycoprotein and is expressed on plasma cells, but also on other hematopoietic cells. Anti-CD38 monoclonal antibodies have changed the treatment landscape of multiple myeloma in the last years. Daratumumab and Isatuximab have been approved in different combinations and lines of therapy for multiple myeloma and are broadly used [111]. Preclinical studies demonstrated efficacy of CD38-directed CAR-T cells, ADCs, and bispecific antibodies, but to date no clinical data are available [112,113,114]. Data from a phase I study of a bispecific CAR-T cell construct targeting CD38 and BCMA showed an ORR of 87.5%. Toxicities were manageable and responses were ongoing up to 51 weeks [115].

### 5.5. Dual Targeted CAR-T Cells

Several studies are currently evaluating the simultaneous targeting of two different antigens to improve responses and avoid acquired resistance. These strategies combine mostly BCMA with another antigen such as CD19, SLAMF7, or CD38, either through co-infusion of two different CAR-T cell products or with dual-targeted CAR-T cells [115,116,117].

An ongoing first-in-human study is investigating a dual FasT CAR-T cell therapy targeting BCMA and CD19 simultaneously. The data of 16 heavily pretreated patients with multiple myeloma were presented. The median number of prior lines was five. All patients developed CRS; two patients had grade 3 CRS, while all others were grade 1 or 2. No neurotoxicity was observed. Patients in all dose levels responded, and the ORR was 93.8%. At data cut-off, the best response was MRD-negative CR/sCR in 9/16 patients at a median follow up of 7.3 months [116].

### 5.6. Other Targets

Other potential targets for the treatment of multiple myeloma are evaluated in preclinical and very early clinical settings. These include CD138, CD229, CD44v6, and CD46, among others [118,119,120,121].

CD138 (or syndecan-1) is expressed on normal and malignant plasma cells and therefore seems to be a reliable target for multiple myeloma. CD138-specific CAR-T cells were able to eliminate myeloma cells in vitro and in vivo [118]. The first clinical results showed a response in 4 of 5 treated patients, but no durable responses were observed [122].

CD229 CAR-T cells efficiently eliminated differentiated MM plasma cells and also memory B cells, a potential reservoir for MM-propagating cells, in myeloma cell lines and xenograft mouse models [119].

Clinical data of these alternative targets have to be awaited.

## 6. Who Is Who—How to Find the Right BCMA-Targeted Drug for the Right Patient? Potential Key Patient Selection Criteria for ADCs vs. Bispecific Antibodies vs. CAR-T Cells

Since the introduction of numerous new anti-myeloma drugs and treatment concepts such as cellular therapies in recent years, it has become extremely challenging for physicians to address the most appropriate therapy for each individual patient with multiple myeloma.

BCMA is extensively studied and has been approved as a promising target for clonal-directed MM therapies [123]. The three most promising treatment modalities targeting BCMA are bispecific antibodies, antibody–drug conjugates, and CAR-T cell constructs [124,125,126]. To date, Belantamab mafodotin is the only approved BCMA-targeting drug for RRMM. However, there are multiple other targets comprising novel ADCs, CAR-T cells, and bispecific antibodies. A major challenge for the future is to define the most effective therapy combinations and the best sequence of the different substances for the individual patient.

To opt for the best treatment regimen, clinicians have to consider plenty of disease-related and patient-related factors, such as disease morbidity (e.g., refractory disease, renal impairment, extramedullary disease, or aggressiveness of disease in general), age and frailty (performance status, disability), and co-morbidities. Further important tools for decision making are risk assessment (cytogenetics and R-ISS), treatment history (previous therapies), and patient’s preference and social support (family and/or travel support) [127,128]. All these factors should be incorporated into the decision-making process aiming for the most effective treatment regimen which is also safe and well-tolerated and supports good quality of life.

### 6.1. Disease Based Decision Factors

For difficult-to-treat patient subsets, such as extramedullary disease (EMD), plasma cell leukemia, high-risk (HR) cytogenetics or high tumor burden, two CAR-T cell trials have shown efficacy, and moreover, the efficacy was not negatively influenced by these adverse prognostic parameters [22,27,129]. Early-phase trials with bispecific antibody constructs also included patients with high-risk features (such as EMD, HR, or high tumor burden). No large subgroup analyses have been available until now, but preliminary results suggest that these adverse disease-specific factors may not significantly influence efficacy in patients treated with BsAbs [54,57,98,104]. If these findings can be confirmed by extended subgroup analyses, CAR-T cells and bispecific monoclonal antibodies will become the highest priority for high-risk disease.

Another important disease-specific high-risk factor will directly influence the choice of therapy: the intensity of disease aggressiveness. It is important to know that CAR-T cell therapy is not available immediately like an emergency drug. The creation and production of CAR-T cells is highly personalized, patient-specific, and time consuming. Highly specialized multi-disciplinary teams are needed. Leukapheresis and lymphodepletion, and genetic editing are the mainstays of the CAR-T cell manufacturing process. In contrast, bispecific monoclonal antibodies are off the shelf products and probably the preferable strategy in the case of rapidly progressing and aggressive disease. Taken together, the vein-to-vein time may significantly influence the selection of the regimen.

Renal impairment is a frequent problem in multiple myeloma and represents an important factor in treatment-decision making. Most clinical trials including those investigating CAR-T cells and BsAbs only enroll patients with normal renal function or a moderately reduced glomerular filtration rate (creatinine clearance >40–45 mL/min). The Belantamab mafodotin-based DREAMM trials are less restrictive regarding patients with renal dysfunction. The results suggest that this ADC may be suitable for patients with renal impairment.

Very special high-risk situations within the MM entity are plasma cell leukemia (PCL) and CNS involvement. These conditions are also listed as clear exclusion criteria in most of the clinical studies. Therefore, only very limited data are available for these patient subgroups. The use of CAR-T cells in PCL is obvious, considering the good efficacy of this therapy in other aggressive lymphomas and acute lymphoblastic leukemia [9,10,11,12]. In addition, currently available therapeutic options in PCL can often achieve only a short response, making CAR-T cell therapy a potentially attractive alternative. However, generating valid data for this particular entity is difficult due to its rarity.

Having demonstrated promising response rates of BCMA-targeting therapeutics in RRMM, studies are now ongoing or planned in earlier lines of therapy in a wide variety of combinations, including first line combinations, consolidation, and maintenance. It may be beneficial to use T-cell-based or T-cell-interacting agents in earlier lines of therapy to take advantage of better T-cell function. T-cell exhaustion often occurs after multiple therapies, which is a possible explanation for the limited efficacy of these therapeutic approaches in late lines of therapy. Taking this consideration into account, it might be reasonable to collect and store T cells at initial diagnosis. Regardless of an expected good efficacy of a CAR-T cell construct in the setting of first-line therapy in MM, the treatment planning and administration of such a therapy is associated with considerable logistical and financial effort, which makes the use of CAR-T cells for the majority of MM patients probably an unfeasible task. Therefore, it will be interesting to see if similar success can be achieved with BCMA-targeting therapies that are available off-the-shelf.

The data from studies using anti-BCMA therapeutics in early lines of therapy are eagerly awaited. If durable responses can be achieved with the formation of a plateau, then there may also be a possibility to use BCMA-targeting agents in newly diagnosed MM or even in Smoldering MM in the future.

### 6.2. Patient Based Decision Factors

Additional factors with a high impact on the course of disease are the individual patient-based conditions. The most important features are age and frailty. Although there is no age cut-off in cell therapy studies, most patients are fit and do not meet frailty criteria (median age around 61 years in CAR-T cell trials and 64 years in studies with BsAbs) [33,55,62,130,131]. However, in a subgroup analysis with ide-cel patients >65 and >70 years of age, both subgroups had comparable DoR and PFS to the ITT population [23]. These results imply that this patient subgroup of older MM patients achieves the same efficacy and the same safety results, and that CAR-T cells can be applied principally to older but fit patients. For elderly, frail patients with more co-morbidities, presumably T cell engagers (TCE)/bispecific antibodies are more appropriate. More data on this topic with larger patient cohorts have to be analyzed. Other patient-based factors focus on organ function in general, e.g., normal organ function is required for CAR-T cells and TCE, but CAR-T cell therapy requires a more stringent evaluation. In fact, a cardiac and neurologic assessment is required as well as functional respiratory tests and vein access evaluation has to be performed. By contrast, belantamab mafodotin does not require a stringent evaluation except continuous ophthalmological assessment and monitoring.

The familiar and social support has to be considered also [16]: since CAR-T cell therapy requires hospitalization from lymphodepletion for up to 14 days; most protocols require that patients have to stay within a 60-min drive around the hospital after discharge, at least for the first month after CAR-T cell infusion. By contrast, TCEs require hospitalization only at priming doses and until full dosing because of potential CRS development. The continuous treatment can be performed in an outpatient setting. Moreover, ADCs such as Belantamab mafodotin are off-the-shelf and outpatient administration can be performed from the beginning.

Quality of life is a particularly important aspect of any therapy for the patient. Evaluations during and after CAR-T cell therapy with ide-cel and cilta-cel have shown significant improvements in quality of life [132,133].

## 7. Conclusions

One of the most important drivers for treatment selection is efficacy. Although specific data are rapidly increasing, most of them are still preliminary up to now. Nevertheless, the new cell-based immune therapies are already included in the recent myeloma treatment guidelines. At present, these very new options are allocated in the later course of disease and most of the patients have already been exposed to PIs, IMIDs and anti-CD38 antibodies. Treatment efficacy may be increased by bringing the new options into the earlier lines of therapy. Answering this question will be one of the most important issues for the near future.

Taken together, patients with MM will be exposed to IMIDS, PIs and CD38 antibodies within the first or second line of therapy in the near future. The trend towards using all these drugs during the first line/induction therapy will lead to a large cohort of triple-class refractory MM patients early in the course of their disease. Therefore, BCMA-targeted therapies will be indicated at an early time point. For these patients, as well as for patients with later relapses, we have to consider patient and disease-related factors in order to offer the most appropriate therapy to each individual MM patient.

## Figures and Tables

**Table 1:** Characteristics, efficacy and safety data from selected clinical trials of BCMA-targeting CAR-T cell constructs.

CAR-T Cell Construct (Name)	Study Name and/or Phase	Number of Patients	Triple-Class Refractory, %	High-Risk Cytogenetics/EMD, %	Median PFS, Months (95% CI)	Median OS, Months (95% CI)	≥CR, %	CRS, All Grades, %	Neurotoxicity, All Grades, %	NCT Number	References
Ide-cel (bb2121)	CRB-401, Phase 1	62	69	27/37	8.8 (5.9–11.9)	34.2 (19.2–NE)	39	76	36	NCT02658929	[18,19]
Ide-cel (bb2121)	KarMMa, Phase 2	128	84	35/39	8.8 (5.6–11.6)	19.4 (18.2–NE)	33	84	18	NCT03361748	[20,21]
Cilta-cel	LEGEND-2, Phase 1/2	57	NR	NR	20 (10–28)	Not reached, 18-month OS 68% (54–79%)	74	90	1	NCT03090659	[26]
Cilta-cel	CARTITUDE-1, Phase 1b/2	97	88	24/13	22.8 (22.8–NE)	Not reached, 18-month OS 80.9% (71.4–87.6%)	80	95	21	NCT03548207	[27,29]
Orva-cel	EVOLVE, Phase 1/2	62	94	41/23	9.3 in the 300 × 10^6^ group (*n* = 19), not reached in the other groups	NR	36	89	13	NCT03430011	[33]
bb21217	CRB-402, Phase 1	69	64	33/NR	NR, mDOR 17.0 (9.4–NE)	NR	29	70	16	NCT03274219	[24]
NCI CAR-BCMA	Phase 1	24	NR	46/NR	NR, mEFS 31 weeks	NR	8	71	NR	NCT02215967	[13,34]
UPenn CART-BCMA	Phase 1	25	72	96/28	65/57/125 days in cohort 1/2/3	NR	8	88	32	NCT02546167	[35]
P-BCMA-101	PRIME, Phase 1/2	55	60	NR	NR	NR	NR, ≥VGPR: 50 (*n* = 6) *	17 (*n* = 53)	4 (*n* = 53)	NCT03288493	[32]
CT053	LUMMICAR STUDY 2, Phase 1	20	85	55/25	NR	NR	25	79	16	NCT03915184	[36]
ALLO-715	UNIVERSAL, Phase 1	31	NR	48/23	NR	NR	≥VGPR: 40	45	0	NCT04093596	[31]
C-CAR088	Phase 1	23	NR	81/NR	Not reached, 6-month PFS 65.1% (47–90)	NR	44	91	4	NCT03751293 NCT03815383 NCT04322292 NCT04295018	[37]

CAR = chimeric antigen receptor; CI = confidence interval; CR = complete remission; CRS = cytokine release syndrome; EMD = extramedullary disease; mDOR = median duration of response; mEFS = median event free survival; NE = not estimable: NR = not reported; OS = overall survival; PFS = progression free survival; VGPR = very good partial remission. * with modified manufacturing process. Only data of studies with results for at least 20 patients are reported.

**Table 2:** Characteristics, efficacy and safety data from selected clinical trials of bispecific T-cell engaging constructs targeting BCMA and CD3.

Agent	Drug Design	Trial Phase	Drug Status	Best ORR %	CRS % (*n*)	NCT Number	References
AMG420	BiTE	1	No further development	70% (at MTD, *n* = 10)	38% (16/42)	NCT03836053	[53]
AMG701	Half-life extended BiTE	1/2	Phase 1/2 study ongoing	83% (last evaluated dose expansion cohort, *n* = 6)	61% (46/75)	NCT03287908	[54]
Teclistamab (JNJ-64007957)	BsAb, IgG4 Fc region (DuoBody)	1/2	Several phase 1/2 studies ongoing, monotherapy and combinations	69% (most active IV and SC doses, *n* = 68)	55% (82/149)	NCT04557098 NCT03145181	[55,56,57,58]
REGN5458	BsAb, IgG4 Fc region (VelociBi)	1/2	Phase 1/2 study ongoing	62.5% (highest tested dose level, *n* = 8)	39% (19/49)	NCT03761108	[59]
TNB-383B	BsAb, IgG4 Fc region, dual BCMA binding domains	1	Phase 1 study ongoing	80% (highest tested dose levels, *n* = 15)	45% (26/58)	NCT03933735	[60]
Elranatamab (PF-06863135)	BsAb, IgG2a Fc region	2	Phase 2 study ongoing (MAGNETISMM-3)	83.3% (RP2D SC, *n* = 6)	73% (22/30)	NCT04649359	[61]
CC-93269	BsAb, IgG1 Fc region, bivalent anti-BCMA arm	1	Phase 1 study ongoing	89% (highest tested dose level, *n* = 9)	77% (23/30)	NCT03486067	[62]

CRS = cytokine release syndrome; IV = intravenous; MTD = maximum tolerated dose; ORR = overall response rate; RP2D = recommended phase 2 dose; SC = subcutaneous.

**Table 3:** Characteristics and drug status of BCMA-targeting antibody drug conjugates.

Agent	Cytotoxic Conjugate	Trial Phase	Drug Status/Published Results	NCT Number	References
Belantamab mafodotin (GSK2857916)	Monomethyl auristatin F (MMAF)	2, 3	First-in-class approval 2020; several studies with different drug combinations ongoing	NCT02064387	[73]
MEDI2228	Pyrrolobenzodiazepine (PBD)	1	Early phase 1 results published	NCT03489525	[74]
HDP-101	Amanitin	1/2	Phase 1 not yet recruiting	NCT04879043	[75]
AMG-224	Mertansine (DM1)	1	Early phase 1 results published; deprioritized in favor of the development of bispecific antibody constructs by the company	NCT02561962	[76]
CC99712	Undisclosed	1	Recruiting, no results available	NCT04036461	
